# Age- and disability-based trends in potentially preventable hospitalizations: evidence from nationwide claims data in Korea

**DOI:** 10.4178/epih.e2026012

**Published:** 2026-02-27

**Authors:** Hyejung Yoon, Boyoung Jeon, Seyune Lee, Daesung Choi, Se-Youn Jung, Dong-Min Son, Yong Joo Rhee, Juhyeon Moon, So Youn Park, In-Hwan Oh, Young-il Jung

**Affiliations:** 1 Department of Environmental Health, Korea National Open University, Seoul, Korea; 2 Department of Health and Medical Information, Myongji College, Seoul, Korea; 3 Hubert Department of Global Health, Rollins School of Public Health, Emory University, Atlanta, GA, USA; 4 Prime College, Korea National Open University, Seoul, Korea; 5 Department of Nursing, Ansan University, Ansan, Korea; 6 Department of Health Science, College of Natural & Information Science, Dongduk Women’s University, Seoul, Korea; 7 Division for Gender-based Violence Research, Korean Women’s Development Institute, Seoul, Korea; 8 Department of Hospital Medicine, Inha University Hospital, Incheon, Korea; 9 Department of Preventive Medicine, University of Ulsan College of Medicine, Seoul, Korea

**Keywords:** Ambulatory care sensitive conditions, Persons with disabilities, Older adults, Claims data, Trends, Diabetes mellitus, Hypertension

## Abstract

**OBJECTIVES:**

Individuals with disabilities are at greater risk of hospitalization than the general population. We examined 10-year trends in potentially preventable hospitalizations (PPH) in Korea, comparing individuals with and without disabilities and assessing age-specific patterns.

**METHODS:**

Using National Health Information Database claims data (2010–2019), we established a fixed cohort of newly registered individuals with disabilities and control subjects statistically matched (1:1.5) at baseline. Annual PPH rates among patients with each condition were calculated and age- and sex-standardized according to Organization for Economic Cooperation and Development Health Care Quality Indicators definitions. Trends and annual percent changes (APCs) were analyzed by disability status and age group (non-older: 30–64; older adults: ≥65 years).

**RESULTS:**

Between 2010 and 2019, PPH rates declined significantly in both groups. Among individuals with disabilities, the steepest decline was observed for hypertension (APC, −15.7%; 95% confidence interval [CI], −17.7 to −13.7), whereas congestive heart failure showed the largest reduction among individuals without disabilities (APC, −7.8%; 95% CI, −10.8 to −4.7). Declines were generally greater among non-older adults aged 30–64 years, regardless of disability status. The disparity between disability and non-disability groups narrowed over the decade, primarily due to larger improvements among non-older adults. Older adults with disabilities consistently exhibited the highest PPH rates for most conditions, whereas younger individuals with disabilities had the highest rates for diabetes.

**CONCLUSIONS:**

PPH rates declined over the decade among both individuals with and without disabilities, particularly for hypertension and among non-older adults. However, older adults with disabilities remain at elevated risk, underscoring the need for targeted strategies to improve access to community-based primary care.

## GRAPHICAL ABSTRACT

[Fig f3-epih-48-e2026012]

## Key Message

This study examined trends in potentially preventable hospitalizations (PPH) in Korea from 2010 to 2019, comparing individuals with and without disabilities. Overall, PPH rates declined in both groups, with the largest reductions observed for hypertension and among adults aged 30–64 years. Disability-related disparities also narrowed over time; however, they persisted, particularly for diabetes and among older adults with disabilities. Despite narrowing disparities, the persistently high PPH risk among older adults with disabilities underscores the urgent need for targeted strategies to enhance access to community-based primary care.

## INTRODUCTION

The number of individuals with disabilities has increased due to longer life expectancy and the growing prevalence of chronic diseases. It is estimated that 1 in 6 people worldwide lives with a disability [1]. This demographic shift has intensified demand for health and social services, particularly medical care. Compared with individuals without disabilities, those with disabilities experience greater vulnerability in both socioeconomic and health domains. They have a higher prevalence of chronic conditions—averaging 3.4 per person compared with 2.4 among those without disabilities [2]—and report higher levels of unmet health care needs [3]. Their patterns of health care utilization also differ, characterized by lower use of preventive services, higher inpatient and outpatient utilization, and greater medical expenditures [2,4,5]. Collectively, these patterns suggest persistent barriers to accessing high-quality care among individuals with disabilities.

A key indicator of access to and quality of community-based primary care is potentially preventable hospitalization (PPH). PPH refers to hospital admissions for specific conditions—commonly known as ambulatory care–sensitive conditions—that could generally be avoided with timely and effective outpatient care [6]. The PPH index is included in the Health Care Quality Indicators (HCQI) developed by the Organization for Economic Cooperation and Development (OECD) [7] and is used in several countries, including the United States and Canada, to support performance-based payment systems [6,8-10]. The specific conditions included in PPH indicators vary slightly across settings [6,7,11,12]. In the OECD HCQI framework, PPH is defined for 5 major conditions: hypertension (HTN), diabetes mellitus (DM), asthma, chronic obstructive pulmonary disease (COPD), and congestive heart failure (CHF) [7].

PPH has been widely studied as an indicator of access to and quality of primary care. Prior research has examined risk factors for PPH [13,14] and documented disparities across regions [15-17], countries [18], and socioeconomic groups within populations [19-22]. Risk factors have been most extensively investigated among high-risk groups, such as individuals with chronic conditions and older adults. In general, individuals at higher risk of PPH tend to have lower socioeconomic status (e.g., lower education and income), belong to racial or ethnic minority groups [23], have a history of chronic illness [24], or face barriers to preventive care, such as limited access to annual wellness visits [25,26].

Despite representing a highly vulnerable population, individuals with disabilities remain under-represented in PPH research compared with the extensive literature on populations defined by race or socioeconomic status [19-23]. This gap likely reflects the substantial heterogeneity in disability type, severity, and duration [27,28]. Existing studies have primarily focused on specific disability categories [25,29-33] or individual diseases [19,20,33], rather than examining the population of individuals with disabilities as a whole. Nevertheless, individuals with disabilities are consistently found to be at higher risk of hospitalization than the general population. A comprehensive analysis of trends and disparities in PPH between individuals with and without disabilities is therefore necessary to inform strategies aimed at improving primary care access and advancing health equity.

This study compared PPH rates in Korea between individuals with and without disabilities over the past decade using a retrospective cohort derived from the National Health Insurance Service (NHIS) claims database. Particular attention was given to age-specific patterns, as the growing population of older adults with disabilities faces the dual challenges of aging and disability, both of which may hinder access to health care. Accordingly, subgroup analyses were conducted for individuals aged 30–64 years and those aged ≥65 years.

## MATERIALS AND METHODS

### Study design and population

This study was a descriptive trend analysis using nationwide, population-based data from 2010 to 2019 extracted from the NHIS claims database. To identify newly registered individuals, we applied a 2-year washout period (2008–2009). Individuals newly registered with a disability between 2010 and 2019 were identified, and comparison subjects without disabilities were selected at a 1:1.5 ratio using 1-time, static propensity score matching. Matching was performed at baseline (with 2010 as the reference year) to establish a fixed study population based on age, sex, place of residence, and National Health Insurance premium quintile.

Based on the characteristics of the 5 chronic conditions included in the OECD HCQI 2016–2017 framework [34], the analytic sample was restricted to individuals aged ≥30 years. Individuals with internal organ disorders, facial disorders, or epilepsy were excluded to minimize potential confounding and diagnostic overlap, as these disability types are intrinsically linked to health care utilization and conditions included in the PPH indicators [20,35,36]. Individuals missing information on disability type or severity after initial registration, as well as those with incomplete premium data, were also excluded. The final analytic dataset included 742,287 individuals with disabilities (3,442,210 observations) and 1,290,681 individuals without disabilities (12,334,419 observations) across the study period.

**Figure 1.**
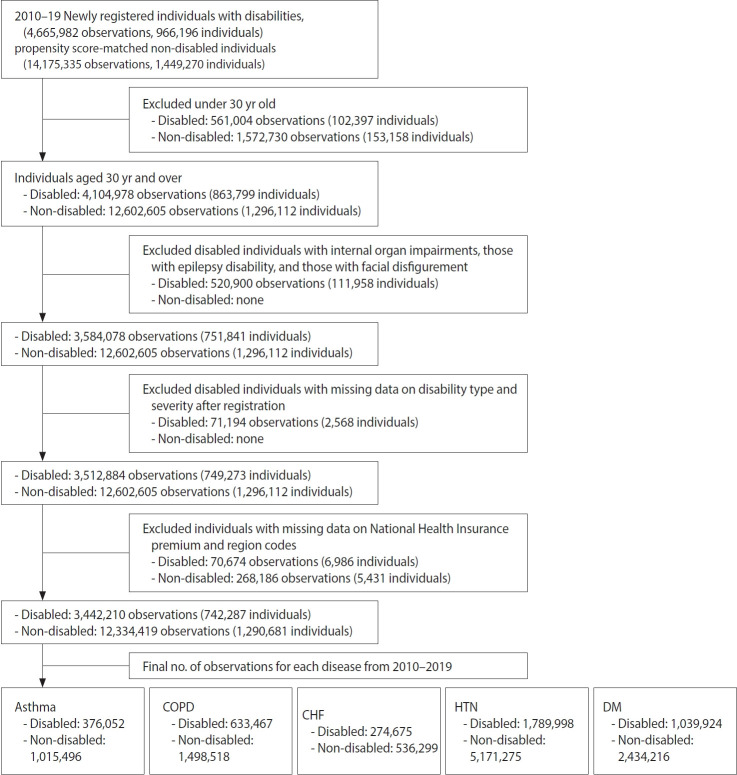
Flow diagram of study population. COPD, chronic obstructive pulmonary disease; CHF, congestive heart failure; HTN, hypertension; DM, diabetes mellitus.

From this fixed analytic dataset, condition-specific patient groups corresponding to PPH were identified annually according to the OECD HCQI 2016–2017 definitions [34]. For each year, individuals with asthma, COPD, CHF, HTN, or DM were identified (Supplementary Material 1). Patients were defined as having a condition if they had at least 2 outpatient visits or at least 1 hospitalization for the same condition within that year. Diagnoses were identified using the International Classification of Diseases, 10th revision; both primary and secondary diagnoses were included. Although the base cohort was fixed at 2010, the analytic subsample for each condition was identified annually based on patients’ clinical status in the respective year. To account for mortality, the patient group for each year included only individuals who were alive and met the diagnostic criteria during that year. The final analytic sample by condition is presented in Figure 1.

### Measures

PPH was defined as an admission with a primary diagnosis matching the disease codes specified in the OECD HCQI 2016–2017 framework [34]. Following HCQI inclusion and exclusion criteria, admissions involving same-day discharge, in-hospital death, or hospitalization related to pregnancy, delivery, puerperium, neonatal care, or newborn conditions were excluded. For each condition, PPH was coded as 1 if a patient experienced ≥1 preventable hospitalization within a given year and 0 otherwise.

For each condition, the annual PPH rate was calculated as the proportion of patients with ≥1 preventable hospitalization among all patients diagnosed with that condition in a given year. To account for differences in demographic structure between the disability and non-disability groups, all results are presented as age- standardized and sex-standardized annual rates.

For age–sex standardization, individuals aged ≥50 years were classified into 5-year age bands, whereas those aged 30–49 years were combined into a single category because of the low frequency of PPH events in this range. For subgroup analyses, age was dichotomized as 30–64 years and ≥65 years. Health insurance coverage was recoded into 2 categories: National Health Insurance and Medical Aid.

### Statistical analysis

General characteristics of the study population were described using participants in 2019. Age-standardized and sex-standardized PPH rates by condition were calculated annually using the 2019 resident registration population aged ≥30 years as the standard population.

To assess differences between individuals with and without disabilities, we quantified annual disparities using both the rate difference (RD) and the rate ratio (RR). Longitudinal trends from 2010 to 2019 were assessed by estimating annual percent change (APC). For PPH rates and RRs, we applied log-linear regression to estimate APCs; for RDs, we used simple linear regression to estimate the absolute annual change. Calendar year was modeled as a continuous variable in all trend analyses. All data extraction, management, and statistical analyses were performed using SAS version 9.4 (SAS Institute Inc., Cary, NC, USA). Standardized rates were calculated using Microsoft Excel (Microsoft, Redmond, WA, USA).

### Ethics statement

This study was conducted in accordance with the Declaration of Helsinki. It used nationwide, population-based data provided by the NHIS in anonymized and de-identified form, in accordance with the Regulations on the Provision and Operation of National Health Information Data. Because the study met the criteria for a waiver of informed consent, ethical approval, including the waiver of informed consent, was granted by the Institutional Review Board of Kyung Hee University (No. KHSIRB-21-330(EA)).

## RESULTS

### Baseline characteristics of the study population in 2019

Table 1 summarizes the baseline characteristics of individuals with and without disabilities across 5 conditions: asthma, COPD, CHF, HTN, and DM. In 2019, 36–40% of patients across these conditions had a disability. Sex distributions differed by condition: patients with COPD and DM were more frequently male in both groups (p<0.001), whereas individuals with disabilities were more frequently female among those with asthma, CHF, and HTN (all p<0.001). Across all conditions, the majority of patients were aged ≥65 years, and the proportion aged 30–49 years was the smallest; however, this younger age group constituted a relatively larger share among individuals with disabilities. Medical Aid coverage was consistently more prevalent among individuals with disabilities across all 5 conditions (all p<0.001).

### Potentially preventable hospitalization status in 2019

**Figure 2.**
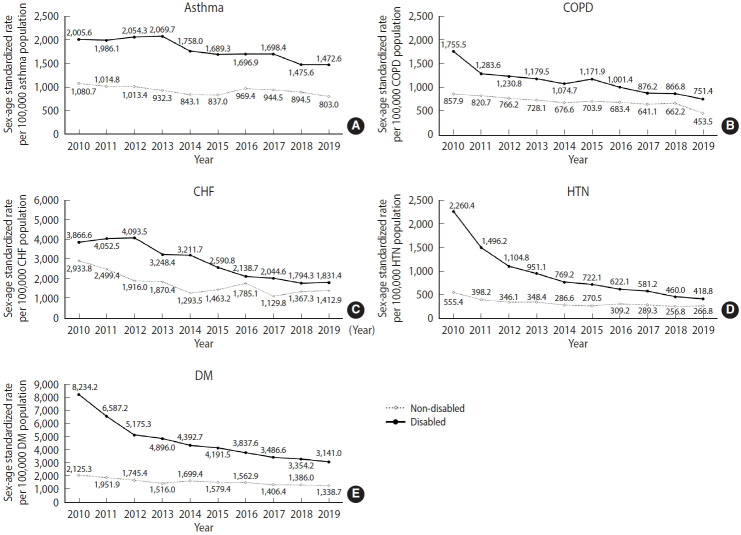
Sex-age standardized rate1 of potentially preventable hospitalization from 2010 to 2019 (A: asthma, B: COPD, C: CHF, D: HTN, E: DM). PPH, potentially preventable hospitalization; COPD, chronic obstructive pulmonary disease; CHF, congestive heart failure; HTN, hypertension; DM, diabetes mellitus. 1Standardized rates were calculated per 100,000 population for each specific condition; The rates were age- and sex-standardized using the 2019 Korean population aged 30 years and older as the standard population.

Figure 2 presents age-standardized and sex-standardized PPH rates from 2010 to 2019. In 2019, among individuals with disabilities, DM had the highest rate (3,141.0 per 100,000 DM patients; 95% confidence interval [CI], 2,974.2 to 3,307.9), whereas HTN had the lowest rate (418.8; 95% CI, 366.2 to 471.5). In contrast, among individuals without disabilities, CHF had the highest rate (1,412.9; 95% CI, 1,031.6 to 1,794.2), and HTN had the lowest (266.8; 95% CI, 214.9 to 318.8).

The largest disparity between individuals with and without disabilities was observed for DM, with an RD of 1,802.3 per 100,000 patients and an RR of 2.35 (3,141.0 vs. 1,338.7 per 100,000 patients).

When stratified by age, disease-specific disparities varied; however, overall gaps were greater among non-older adults than among older adults. In the non-older group, PPH rates among individuals with disabilities ranged from 1.4-fold higher for CHF to 2.6-fold higher for DM compared with those without disabilities. Among older adults, the corresponding disparities ranged from 1.1-fold (CHF) to 1.6-fold (DM) (Table 2).

### Potentially preventable hospitalization trends, 2010–2019

The annual distribution of the study population and the number of individuals with ≥1 PPH across the 5 conditions are presented in Supplementary Materials 2 and 3. Overall, although the number of patients with specific chronic conditions increased—particularly among individuals with disabilities—the number of preventable hospitalizations did not increase proportionally, resulting in a consistent decline in standardized PPH rates. For example, the number of individuals with disabilities and DM increased approximately 8-fold from 2010 to 2019 (from 26,153 to 209,502); however, the number of patients with ≥1 PPH increased only 3.2-fold during the same period (from 1,760 to 5,647).

Table 2 summarizes PPH rates at 4 time points (2010, 2013, 2016, and 2019) and reports APCs for each disability and age group. In addition, Table 2 presents disparities between individuals with and without disabilities, quantified using RD and RR, along with corresponding APCs to assess changes in these gaps over time.

### Overall trends by disability status

Trend analyses demonstrated statistically significant declines in PPH rates among both individuals with and without disabilities. Among individuals with disabilities, HTN-related PPH exhibited the steepest decline (APC, −15.7%; 95% CI, −17.7 to −13.7), whereas among individuals without disabilities, CHF showed the largest reduction (APC, −7.8%; 95% CI, −10.8 to −4.7). In contrast, asthma exhibited the smallest decline in both groups (APC, −3.8%; 95% CI, −4.8 to −2.9 among individuals with disabilities; APC, −2.3%; 95% CI, −3.6 to −0.9 among individuals without disabilities).

### Trends in disparities between individuals with and without disabilities

Over the decade, absolute disparities (RD) between individuals with and without disabilities narrowed for all conditions except CHF, for which no statistically significant reduction was observed. The greatest improvement was observed for DM, with the RD decreasing by 409.5 per 100,000 patients annually (APC, −410.0; 95% CI, −524.7 to −294.3), followed by HTN and COPD. In contrast, asthma-related disparities showed the smallest improvement, with an annual RD reduction of 47.0 per 100,000 patients (APC, −47.0; 95% CI, −67.5 to −26.6) (Table 2).

### Age-specific trends by disability status

When stratified by age, downward trends in PPH rates were observed in most groups regardless of disability status. However, among individuals without disabilities aged ≥65 years, reductions in asthma-related and CHF-related PPH were not statistically significant. Overall, non-older adults experienced more pronounced declines than older adults, with 1 exception: for asthma among individuals with disabilities, the decline was slightly steeper among older adults (APC, −4.0%; 95% CI, −5.4 to −2.5) than among non-older adults (APC, −3.8%; 95% CI, −5.1 to −2.5). Among individuals with disabilities, HTN exhibited the steepest 10-year decline across all age groups. In contrast, among individuals without disabilities, the largest reduction was observed for CHF in the non-older group and for HTN in the older adult group. Across all age categories, individuals with disabilities demonstrated steeper declines in PPH rates than their counterparts without disabilities (Table 2).

### Age-specific trends in disparities

Patterns of disparity reduction varied by age group. Among older adults, absolute disparities (RD) declined significantly across all 5 conditions, with the largest reduction observed for DM, followed by CHF, HTN, asthma, and COPD. Similarly, among non-older adults, DM exhibited the greatest annual decrease in absolute disparity, followed by HTN, COPD, and asthma. However, CHF-related disparities among non-older adults did not decline significantly during the study period (Table 2).

In summary, age-standardized and sex-standardized PPH rates for all 5 conditions declined between 2010 and 2019, accompanied by a narrowing of disparities between individuals with and without disabilities. Although the overall downward trend was consistent, the magnitude of reduction varied by condition and was more heterogeneous in age-stratified analyses (Supplementary Materials 2 and 3). Across most chronic conditions, individuals with disabilities consistently experienced higher PPH rates than those without disabilities, with the highest rates observed among adults aged ≥65 years. However, DM represented a notable exception and the most pronounced disparity: it was the only condition for which the highest PPH rates were observed among individuals with disabilities aged <65 years rather than among older adults (Supplementary Materials 2 and 3).

## DISCUSSION

This study used nationwide NHIS claims data to examine 10-year trends (2010–2019) in PPH among individuals with and without disabilities. Subgroup analyses were conducted by age (<65 and ≥65 years) to identify age-specific patterns. The main findings can be summarized as follows.

First, PPH rates declined over the past decade in both disability and non-disability groups across all 5 conditions, with the greatest reduction observed for HTN. Age-stratified analyses indicated that declines were more pronounced among adults aged <65 years, particularly for HTN-related PPH among individuals with disabilities.

Second, disparities in PPH between individuals with and without disabilities narrowed during the study period. The greatest reduction in absolute disparities was observed for DM, whereas asthma showed the smallest improvement. In both younger and older age groups, DM exhibited the largest decrease in disability-related disparities.

Third, despite these improvements, substantial differences persist, with DM representing the most pronounced inequity. Individuals with disabilities continued to experience higher PPH rates, particularly those aged ≥65 years, who constituted the highest-risk group for most conditions. Notably, DM-related PPH was especially prevalent among non-older adults with disabilities.

The observed decline in PPH is consistent with findings from prior research [20,37,38]. Although prevalence estimates vary depending on the study population, case definitions, observation period, and analytic approach, the overall downward trend has been consistently reported. In the present study, age-standardized and sex-standardized rates were somewhat higher than those reported in previous Korean studies [20,37]. This difference is likely attributable to differences in study populations: our analysis was restricted to adults aged ≥30 years with pre-existing diagnoses of the relevant conditions, whereas prior studies included the general adult population aged ≥15 years [20] or ≥19 years [37].

Previous research suggests that PPH is influenced by a range of individual-level and regional-level factors, and the recent decline observed in this study likely reflects multiple concurrent influences. In the Korean context, 2 factors may be particularly relevant: reductions in unmet health care needs and increases in outpatient utilization among individuals with disabilities.

National survey data indicate that unmet health care needs have declined over time in both the general population and among individuals with disabilities [39,40]. In addition, health insurance statistics show that the average number of outpatient visits among individuals with disabilities increased between 2016 and 2019 [36]. Although this study did not directly evaluate the associations between outpatient utilization, unmet needs, and PPH, improved access to high-quality ambulatory care among socially vulnerable populations may plausibly contribute to reductions in preventable hospitalizations. Future research should more directly examine these mechanisms in populations with disabilities, particularly how improvements in outpatient utilization and reductions in unmet health care needs translate into lower PPH risk.

Korea has invested substantial resources in chronic disease management, particularly for HTN and DM. The marked reductions in HTN-related and DM-related PPH are often attributed to community-based management initiatives [41]; however, empirical evidence directly confirming this relationship remains limited. Furthermore, the extent of participation by individuals with disabilities in these nationwide programs has not been well documented. Individuals with disabilities may face barriers to participating in such initiatives, underscoring the need for targeted support and ongoing evaluation of program impacts within this population.

Disparities in PPH between individuals with and without disabilities were more pronounced among adults aged <65 years. Individuals with disabilities are known to experience accelerated aging compared with their non-disabled counterparts [1,4], suggesting that younger adults with disabilities may exhibit health care utilization patterns similar to those of older adults. In Korea, many health care policies and initiatives are targeted toward populations aged ≥65 years. This policy focus may partly explain the relatively smaller disparities observed among older adults compared with younger adults. In addition, non-older adults with disabilities may encounter limited access to public health services, which could contribute to unmet care needs. This pattern is particularly evident for DM, where the physiological burden associated with disability, combined with limited targeted management for younger adults, may contribute to the elevated PPH rates observed in this study. Unlike older adults who may benefit from age-specific chronic disease programs, non-older adults with disabilities may fall into a “service gap,” potentially resulting in suboptimal diabetes control and subsequent hospitalization.

The observed reduction in PPH disparities may also be associated with health management policies targeting individuals with disabilities in Korea. The Korean government has implemented several initiatives to improve the health status of individuals with disabilities and to enhance their access to medical care. In 2017, the Act on Guarantee of the Right to Health and Access to Medical Services for Persons with Disabilities was enacted to strengthen chronic disease management in this population. Under this legislation, a family doctor pilot program for individuals with disabilities has been operating since 2018 [42], and the Sixth Comprehensive Plan for Persons with Disabilities was implemented in 2023. These initiatives emphasize community-based chronic disease management and preventive care, with the overarching goal of promoting equitable access to health services.

Evidence from prior studies suggests that public health policies can reduce preventable hospitalizations among individuals with disabilities [43-46]. To achieve sustained reductions, policies should extend beyond expanding coverage to actively encourage participation by primary care providers and develop appropriate incentive structures for both providers and individuals with disabilities [44]. Current governmental efforts to expand disability-related health initiatives represent important progress; however, broader inclusion of target populations and stronger engagement of primary care providers will likely be necessary to further improve access and chronic disease management. Expanding eligibility to encompass all individuals with disabilities and strengthening provider engagement could enhance access to primary care and improve long-term outcomes.

Finally, this study confirmed that DM-related PPH remains disproportionately high, particularly among individuals with disabilities. Within this group, adults aged <65 years emerged as a key at-risk population. Although both DM and HTN have long been central to chronic disease management programs in Korea, the impact of these initiatives appears less pronounced for DM than for HTN [47]. These findings underscore the need to strengthen DM management within community-based primary care, with particular attention to non-older adults with disabilities.

This study compared 10-year trends in PPH between individuals with and without disabilities and conducted age-stratified analyses. To reduce variability related to disability onset and duration [27,28], we included only individuals newly registered with a disability from 2010 onward and excluded those with internal organ disabilities, who have distinct patterns of medical utilization. Several limitations warrant consideration. First, despite restricting the sample to newly registered individuals, the disability group remained inherently heterogeneous. Patterns of health care utilization may vary substantially according to disability type, severity, and the presence of multiple disabilities. We excluded individuals with internal organ disorders to minimize confounding with PPH indicator conditions; however, this exclusion may have led to underestimation of the overall PPH burden among individuals with disabilities. In sensitivity analyses that included internal organ disorders, PPH rates were substantially higher—particularly for COPD, CHF, and DM—although overall longitudinal trends and comparisons with individuals without disabilities remained consistent with the primary findings (Supplementary Material 4). Second, the study period was limited to 2010–2019. The COVID-19 pandemic, which began in 2020, substantially altered health care utilization patterns, including PPH, and disproportionately affected vulnerable populations such as individuals with disabilities [48,49]. Future studies should extend the observation period to include post-pandemic years in order to provide a more comprehensive understanding of evolving disparities in PPH. Third, the comparison group was established through 1-time static matching at baseline (2010) rather than annual dynamic re-matching. Although this approach ensured longitudinal stability of the fixed cohort, it may not have fully captured time-varying changes in socioeconomic characteristics, such as shifts in health insurance premium quintiles, over the 10-year follow-up. Despite efforts to minimize baseline divergence by focusing on newly registered individuals, residual confounding due to changes in social determinants of health may persist and should be considered when interpreting long-term trends. Fourth, clinical comorbidities were not explicitly adjusted for in the matching or standardization procedures. Consistent with our rationale for excluding internal organ disabilities to minimize diagnostic overlap, we chose not to adjust for comprehensive comorbidity indices to avoid potential overadjustment. Because PPH represents a downstream consequence of inadequately managed chronic conditions, adjusting for comorbidity burden could obscure disparities in access to care that this study sought to evaluate. Nevertheless, residual confounding remains possible, and findings should therefore be interpreted with appropriate caution.

## Figures and Tables

**Figure f3-epih-48-e2026012:**
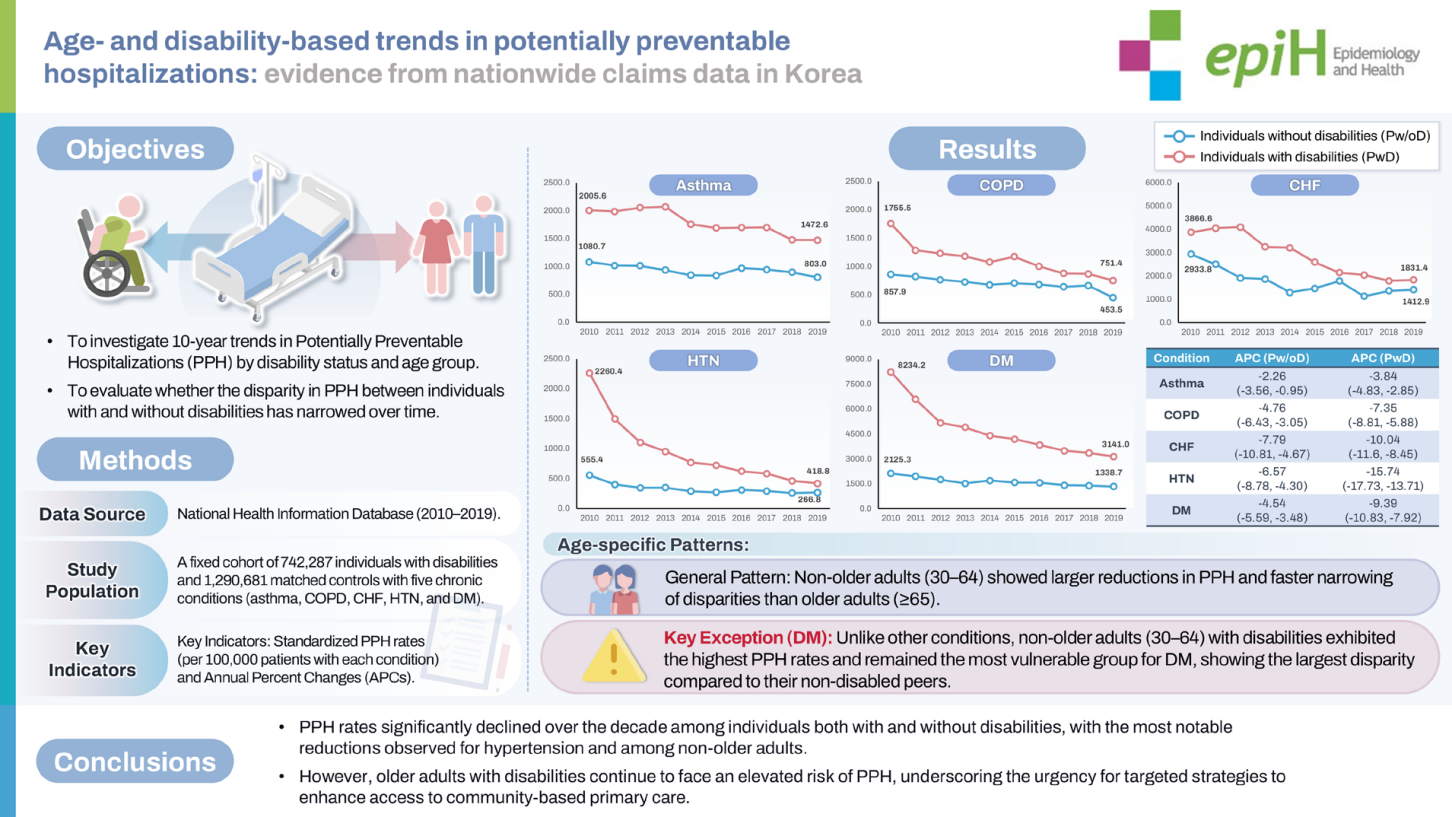


**Table 1.** Baseline characteristics of the study population in 2019

Characteristics	Asthma		COPD		CHF		HTN		DM	
	Pw/oD	PwD	Pw/oD	PwD	Pw/oD	PwD	Pw/oD	PwD	Pw/oD	PwD
Total (n)	120,838	73,782	194,235	126,842	97,319	66,050	620,184	351,944	330,176	212,691
Sex										
Male	50.9	48.8	53.8	52.7	50.4	49.1	51.0	49.4	54.9	52.6
Female	49.1	51.2	46.2	47.3	49.6	50.9	49.0	50.6	45.1	47.4
Age (yr)										
30–49	4.8	5.6	4.3	6.0	1.0	3.1	2.0	4.1	2.4	5.3
50–64	14.9	17.5	15.4	19.5	8.6	14.6	17.1	21.3	18.6	23.6
≥65	80.3	76.9	80.3	74.6	90.4	82.3	80.8	74.6	79.1	71.1
Premium quintile^1^										
0 (Medical Aid)	7.9	15.9	7.8	16.4	9.0	15.4	6.3	14.1	6.9	16.4
1 (lowest)	17.1	16.5	17.2	16.5	16.5	16.1	17.5	17.0	17.2	16.8
2	14.2	13.4	14.5	13.5	13.6	12.9	14.7	14.1	14.6	13.9
3	20.8	19.1	20.8	19.2	19.7	18.6	20.9	19.4	20.8	19.2
4 (highest)	39.9	35.2	39.8	34.4	41.2	37.0	40.7	35.5	40.5	33.8

Values are presented as column %. PwD, persons with disabilities; Pw/oD, persons without disabilities; COPD, chronic obstructive pulmonary disease; CHF, congestive heart failure; HTN, hypertension; DM, diabetes mellitus.

1 The premium quintile is expressed as a percentage of the total, excluding missing values.

**Table 2.** Change in the age- and sex-standardized rate^1^ of PPH, 2010–2019

Variables	2010	2013	2016	2019	APC (95% CI)	p-value
Asthma						
All ages						
Pw/oD	1,080.7	932.3	969.4	803.0	–2.26 (–3.56, –0.95)	<0.001
PwD	2,005.6	2,069.7	1,696.9	1,472.6	–3.84 (–4.83, –2.85)	<0.001
RD	924.9	1,137.5	727.5	669.6	–47.01 (–67.46, –26.55)	<0.001
RR	1.86	2.22	1.75	1.83	–1.59 (–3.10, –0.06)	0.042
Aged 30–64						
Pw/oD	953.4	764.0	799.9	651.1	–3.30 (–4.80, –1.77)	<0.001
PwD	1,946.9	2,097.8	1,665.5	1,428.7	–3.80 (–5.17, –2.42)	<0.001
RD	993.5	1,333.8	865.6	777.6	–39.10 (–66.19, –12)	0.005
RR	2.04	2.75	2.08	2.19	–0.51 (–2.49, 1.51)	0.618
Aged ≥65						
Pw/oD	1,535.6	1,533.5	1,574.4	1,345.4	–0.30 (–1.73, 1.14)	0.679
PwD	2,215.3	1,969.7	1,808.9	1,629.3	–3.96 (–5.00, –2.92)	<0.001
RD	679.7	436.1	234.5	283.9	–75.27 (–104.44, –46.10)	<0.001
RR	1.44	1.28	1.15	1.21	–3.59 (–5.18, –1.99)	<0.001
COPD						
All ages						
Pw/oD	857.9	728.1	683.4	453.5	–4.76 (–6.43, –3.05)	<0.001
PwD	1,755.5	1,179.5	1,001.4	751.4	–7.35 (–8.81, –5.88)	<0.001
RD	897.7	451.4	318.0	297.9	–52.62 (–75.58, –29.67)	<0.001
RR	2.05	1.62	1.47	1.66	–2.52 (–4.40, –0.60)	0.010
Aged 30–64						
Pw/oD	599.6	480.8	430.1	221.8	–7.77 (–10.25, –5.21)	<0.001
PwD	1,560.5	960.1	755.8	550.3	–8.86 (–10.95, –6.72)	<0.001
RD	961.0	479.3	325.6	328.5	–47.36 (–77.69, –17.02)	0.002
RR	2.60	2.00	1.76	2.48	–0.68 (–4.11, 2.86)	0.702
Aged ≥65						
Pw/oD	1,780.4	1,611.4	1,587.8	1,280.9	–1.76 (–3.18, –0.31)	0.017
PwD	2,452.0	1,969.7	1,808.9	1,629.3	–4.93 (–5.87, –3.98)	<0.001
RD	671.6	358.2	221.1	348.4	–71.44 (–92.53, –50.35)	<0.001
RR	1.38	1.22	1.18	1.15	–3.19 (–4.29, –2.08)	<0.001
CHF						
All ages						
Pw/oD	2,933.8	1,870.4	1,785.1	1,412.9	–7.79 (–10.81, –4.67)	<0.001
PwD	3,866.6	3,248.4	2,138.7	1,831.4	–10.04 (–11.60, –8.45)	<0.001
RD	932.7	1,378.0	353.6	418.6	–137.5 (–236.08, –38.93)	0.006
RR	1.32	1.74	1.20	1.30	–2.34 (–6.78, 2.31)	0.318
Aged 30–64						
Pw/oD	2,980.3	1,688.6	1,565.5	1,132.3	–10.96 (–15.25, –6.45)	<0.001
PwD	3,436.4	3,063.1	1,842.2	1,573.1	–11.42 (–13.65, –9.14)	<0.001
RD	456.1	1,374.4	276.7	440.8	–105.83 (–236.14, 24.47)	0.111
RR	1.15	1.81	1.18	1.39	–0.1 (–6.71, 6.97)	0.976
Aged ≥65						
Pw/oD	2,768.0	2,519.8	2,569.8	2,414.9	–0.62 (–1.66, 0.43)	0.244
PwD	5,403.1	3,910.6	3,197.7	2,754.1	–6.78 (–7.54, –6.02)	<0.001
RD	2,635.1	1,390.8	628.0	339.2	–250.62 (–293.51, –207.72)	<0.001
RR	1.95	1.55	1.24	1.14	–6.16 (–7.10, –5.21)	<0.001
HTN						
All ages						
Pw/oD	555.4	348.4	309.2	266.8	–6.57 (–8.78, –4.30)	<0.001
PwD	2,260.4	951.1	622.1	418.8	–15.74 (–17.73, –13.71)	<0.001
RD	1,705.0	602.7	312.8	152.0	–142.27 (–185.17, –99.37)	<0.001
RR	4.07	2.73	2.01	1.57	–9.79 (–10.78, –8.79)	<0.001
Aged 30–64						
Pw/oD	519.5	303.9	258.4	237.9	–7.35 (–10.25, –4.35)	<0.001
PwD	2,333.6	931.6	562.4	391.7	–16.73 (–18.89, –14.52)	<0.001
RD	1,814.1	627.7	304.0	153.8	–151.19 (–198.09, –104.3)	<0.001
RR	4.49	3.07	2.18	1.65	–10.11 (–11.75, –8.44)	<0.001
Aged ≥65						
Pw/oD	683.8	507.4	491.0	370.2	–4.84 (–6.07, –3.60)	<0.001
PwD	1,999.0	1,020.7	835.2	515.7	–12.48 (–14.05, –10.88)	<0.001
RD	1,315.3	513.3	344.2	145.5	–110.41 (–140.35, –80.47)	<0.001
RR	2.92	2.01	1.70	1.39	–7.94 (–8.94, –6.93)	<0.001
DM						
All ages						
Pw/oD	2,125.3	1,516.0	1,562.9	1,338.7	–4.54 (–5.59, –3.48)	<0.001
PwD	8,234.2	4,896.0	3,837.6	3,141.0	–9.39 (–10.83, –7.92)	<0.001
RD	6,109.0	3,380.0	2,274.7	1,802.3	–409.55 (–524.75, –294.35)	<0.001
RR	3.87	3.23	2.46	2.35	–5.04 (–6.28, –3.78)	<0.001
Aged 30–64						
Pw/oD	2,163.5	1,454.8	1,512.3	1,294.7	–5.2 (–6.47, –3.91)	<0.001
PwD	8,902.5	5,161.1	4,044.5	3,332.9	–9.58 (–11.17, –7.96)	<0.001
RD	6,739.0	3,706.2	2,532.2	2,038.3	–443.07 (–575.77, –310.37)	<0.001
RR	4.11	3.55	2.67	2.57	–4.57 (–6.03, –3.09)	<0.001
Aged ≥65						
Pw/oD	1,988.8	1,734.3	1,743.5	1,496.2	–2.32 (–3.00, –1.64)	<0.001
PwD	5,847.3	3,949.3	3,098.6	2,455.7	–8.45 (–9.30, –7.60)	<0.001
RD	3,858.5	2,214.9	1,355.1	959.5	–289.81 (–346.30, –233.31)	<0.001
RR	2.94	2.28	1.78	1.64	–6.24 (–7.03, –5.44)	<0.001

PPH, potentially preventable hospitalization; APC, annual percent change; CI, confidence interval; PwD, persons with disabilities; Pw/oD, persons without disabilities; RD, rate difference; RR, rate ratio; COPD, chronic obstructive pulmonary disease; CHF, congestive heart failure; HTN, hypertension; DM, diabetes mellitus.

1 Standardized rates were calculated per 100,000 population for each specific condition; The rates were age- and sex-standardized using the 2019 Korean population aged 30 years and older as the standard population.

## Data Availability

The data used in this study are not publicly available due to strict restrictions imposed by the NHIS. Availability of the data is permitted only for the study that has been approved through the NHIS data provision review process and the National Health Information Data request review committee’s approval.

## Supplementary materials

Supplementary Material 1. Diagnosis codes for asthma, COPD, CHF, HTN, and DM from the OECD 2018-19 Health Care Quality Indicators data collection

Supplementary Material 2. Annual number of patients and sex-age standardized PPH rates among non-disabled individuals by age group and disease type (2010–2019)

Supplementary Material 3. Annual number of patients and sex-age standardized PPH rates among disabled individuals by age group and disease type (2010–2019)

Supplementary Material 4. Sex-age standardized rate of PPH including all disability types from 2010 to 2019
