# Supplementary material for: Age- and disability-based trends in potentially preventable hospitalizations: evidence from nationwide claims data in Korea
Source: Epidemiol Health. 2026 Feb 27;48:e2026012. doi: 10.4178/epih.e2026012 (PMC13219979; doi:10.4178/epih.e2026012)
Supplement: Supplementary Material 1. — Diagnosis codes for asthma, COPD, CHF, HTN, and DM from the OECD 2018-19 Health Care Quality Indicators data collection [file epih-48-e2026012-Supplementary-1.docx]

**Supplementary Material 1: Diagnosis codes for asthma, COPD, CHF, HTN, and DM from the OECD 2018-19 Health Care Quality Indicators data collection**

| **Diseases** | **Diagnosis codes** |
| --- | --- |
| Asthma | J450, J451, J458, J459, J46 |
| COPD | J40*, J410, J411, J418, J42, J430, J431, J432, J438, J439, J440, J441, J448, J449, J47 |
| CHF | I110, I130, I132, I500, I501, I509 |
| HTN | I10, I119, I129, I139 |
| DM | E100, E101, E102, E103, E104, E105, E106, E107, E108, E109,  E110, E111, E112, E113, E114, E115, E116, E117, E118, E119,  E130, E131, E132, E133, E134, E135, E136, E137, E138, E139,  E140, E141, E142, E143, E144, E145, E146, E147, E148, E149 |

* Qualifies only if accompanied by (a) diagnosis code(s) of J41, J43, J44, J47

Note: Codes according to the International Classification of Diseases, 10th revision

COPD: chronic obstructive pulmonary disease, CHF: congestive heart failure, HTN: hypertension, DM: diabetes mellitus
