# Supplementary material for: Age- and disability-based trends in potentially preventable hospitalizations: evidence from nationwide claims data in Korea
Source: Epidemiol Health. 2026 Feb 27;48:e2026012. doi: 10.4178/epih.e2026012 (PMC13219979; doi:10.4178/epih.e2026012)
Supplement: Supplementary Material 2. — Annual number of patients and sex-age standardized PPH rates among non-disabled individuals by age group and disease type (2010–2019) [file epih-48-e2026012-Supplementary-2.docx]

**Supplementary Material 2:** **Annual number of patients and sex-age standardized PPH rates among non-disabled individuals by age group and disease type (2010–2019)**

| Year | Age_  group | Asthma | | | COPD | | | CHF | | | HTN | | | DM | | |
| --- | --- | --- | --- | --- | --- | --- | --- | --- | --- | --- | --- | --- | --- | --- | --- | --- |
|  |  | No. of Patients | PPH ≥ 1 | Standardized rate (95% C.I.) | No. of Patients | PPH ≥ 1 | Standardized rate (95% C.I.) | No. of Patients | PPH ≥ 1 | Standardized rate (95% C.I.) | No. of Patients | PPH ≥ 1 | Standardized rate (95% C.I.) | No. of Patients | PPH ≥ 1 | Standardized rate (95% C.I.) |
| 2010 | total | 78478 | 1043 | 1080.7  (976.9 - 1184.5) | 104462 | 1430 | 857.9  (783.9 - 931.8) | 24921 | 678 | 2933.8  (2145 - 3722.7) | 417300 | 2380 | 555.4  (497.8 - 613) | 171274 | 3247 | 2125.3  (1955.3 - 2295.3) |
|  | <65 years | 28049 | 301 | 953.4  (824.4 - 1082.4) | 37078 | 280 | 599.6  (509.7 - 689.4) | 5073 | 96 | 2980.3  (1972.7 - 3987.8) | 138253 | 588 | 519.5  (446.3 - 592.6) | 61109 | 1124 | 2163.5  (1947.3 - 2379.6) |
|  | ≥65 years | 50429 | 742 | 1535.6  (1421.5 - 1649.6) | 67384 | 1150 | 1780.4  (1674 - 1886.9) | 19848 | 582 | 2768.0  (2534.3 - 3001.8) | 279047 | 1792 | 683.8  (651 - 716.6) | 110165 | 2123 | 1988.8  (1898.7 - 2078.9) |
| 2011 | total | 83334 | 1098 | 1014.8  (911.1 - 1118.4) | 112924 | 1428 | 820.7  (743.3 - 898.2) | 29839 | 790 | 2499.4  (1795.2 - 3203.6) | 437218 | 2107 | 398.2  (350 - 446.4) | 187348 | 3342 | 1951.9  (1790.4 - 2113.4) |
|  | <65 years | 28077 | 263 | 873.4  (744.1 - 1002.8) | 38309 | 267 | 615.5  (519.7 - 711.3) | 5866 | 89 | 2426.1  (1526.7 - 3325.6) | 138344 | 409 | 345.9  (284.7 - 407) | 63962 | 1044 | 1962.9  (1757.4 - 2168.4) |
|  | ≥65 years | 55257 | 835 | 1519.6  (1414.2 - 1624.9) | 74615 | 1161 | 1553.7  (1462.2 - 1645.1) | 23973 | 701 | 2760.9  (2547.9 - 2973.9) | 298874 | 1698 | 585.1  (556.6 - 613.6) | 123386 | 2298 | 1912.5  (1831.2 - 1993.9) |
| 2012 | total | 96138 | 1354 | 1013.4  (914.5 - 1112.2) | 133483 | 1906 | 766.2  (702.6 - 829.8) | 33912 | 921 | 1916  (1397.4 - 2434.6) | 466311 | 2111 | 346.1  (300.7 - 391.4) | 201762 | 3373 | 1745.4  (1584.7 - 1906.2) |
|  | <65 years | 30190 | 275 | 846.8  (723.3 - 970.3) | 43072 | 267 | 499.3  (421.5 - 577.1) | 6102 | 97 | 1687.0  (1025.6 - 2348.5) | 137425 | 334 | 292.3  (234.7 - 349.9) | 64186 | 873 | 1725.6  (1520.9 - 1930.4) |
|  | ≥65 years | 65948 | 1079 | 1608.4  (1510.5 - 1706.3) | 90411 | 1639 | 1719.3  (1634.2 - 1804.5) | 27810 | 824 | 2733.9  (2535.4 - 2932.3) | 328886 | 1777 | 538.1  (512.6 - 563.5) | 137576 | 2500 | 1816.1  (1742.9 - 1889.4) |
| 2013 | total | 95719 | 1309 | 932.3  (836.5 - 1028.1) | 131483 | 1882 | 728.1  (664.4 - 791.8) | 38663 | 1031 | 1870.4 (1274.4 - 2466.4) | 487716 | 2225 | 348.4  (300.2 - 396.6) | 216175 | 3467 | 1516  (1382.7 - 1649.3) |
|  | <65 years | 28220 | 237 | 764.0  (644.2 - 883.7) | 39055 | 241 | 480.8  (402.5 - 559.1) | 6327 | 83 | 1688.6  (927.3 - 2450) | 133160 | 336 | 303.9  (242.5 - 365.3) | 63944 | 821 | 1454.8  (1285.3 - 1624.4) |
|  | ≥65 years | 67499 | 1072 | 1533.5  (1439.5 - 1627.6) | 92428 | 1641 | 1611.4  (1530.9 - 1692) | 32336 | 948 | 2519.8  (2347.1 - 2692.4) | 354556 | 1889 | 507.4  (484.1 - 530.8) | 152231 | 2646 | 1734.3  (1666.3 - 1802.3) |
| 2014 | total | 103139 | 1326 | 843.1  (751.5 - 934.8) | 146617 | 2088 | 676.6  (621.7 - 731.5) | 45304 | 1269 | 1293.5  (937.3 - 1649.6) | 511289 | 2347 | 286.6  (241.9 - 331.2) | 232588 | 3804 | 1699.4  (1532 - 1866.8) |
|  | <65 years | 28672 | 223 | 700.8  (585.8 - 815.8) | 41050 | 264 | 444.1  (376.9 - 511.4) | 6649 | 72 | 954.2  (500.5 - 1408) | 127151 | 252 | 230.7  (173.8 - 287.5) | 63288 | 824 | 1693.0  (1479.5 - 1906.6) |
|  | ≥65 years | 74467 | 1103 | 1351.5  (1268.7 - 1434.2) | 105567 | 1824 | 1506.9  (1434.1 - 1579.6) | 38655 | 1197 | 2505.1  (2346.7 - 2663.6) | 384138 | 2095 | 486.2  (464.7 - 507.8) | 169300 | 2980 | 1722.0  (1657.9 - 1786.1) |
| 2015 | total | 106128 | 1547 | 837  (752.9 - 921) | 152809 | 2496 | 703.9  (645.2 - 762.5) | 51026 | 1512 | 1463.2  (1017 - 1909.5) | 528047 | 2471 | 270.5  (229.1 - 311.9) | 247203 | 4030 | 1579.4  (1417.3 - 1741.6) |
|  | <65 years | 27475 | 223 | 645.5  (540.6 - 750.3) | 39835 | 241 | 429.5  (357.4 - 501.5) | 6950 | 72 | 1146.3  (576.8 - 1715.9) | 125269 | 223 | 214.2  (161.5 - 266.9) | 64070 | 801 | 1556.5  (1349.7 - 1763.4) |
|  | ≥65 years | 78653 | 1324 | 1521.0  (1434.4 - 1607.5) | 112974 | 2255 | 1684.0  (1609.3 - 1758.6) | 44076 | 1440 | 2595.1  (2439.7 - 2750.5) | 402778 | 2248 | 471.7  (451.3 - 492.1) | 183133 | 3229 | 1661.4  (1601.7 - 1721) |
| 2016 | total | 108220 | 1719 | 969.4  (870.5 - 1068.3) | 164420 | 2652 | 683.4  (624.6 - 742.2) | 61403 | 1875 | 1785.1  (1274.8 - 2295.5) | 549340 | 2841 | 309.2  (261.2 - 357.3) | 265576 | 4584 | 1562.9  (1417.6 - 1708.2) |
|  | <65 years | 27630 | 253 | 799.9  (675.7 - 924.2) | 42777 | 250 | 430.1  (357.4 - 502.9) | 8009 | 108 | 1565.5  (913.5 - 2217.4) | 126609 | 255 | 258.4  (197.1 - 319.7) | 66781 | 837 | 1512.3  (1327.1 - 1697.6) |
|  | ≥65 years | 80590 | 1466 | 1574.4  (1487.7 - 1661.2) | 121643 | 2402 | 1587.8  (1517.9 - 1657.7) | 53394 | 1767 | 2569.8  (2422.2 - 2717.3) | 422731 | 2586 | 491.0  (470.7 - 511.2) | 198795 | 3747 | 1743.5  (1684.1 - 1802.9) |
| 2017 | total | 106665 | 1802 | 944.5  (845.3 - 1043.6) | 171193 | 3039 | 641.1  (588.2 - 693.9) | 72423 | 2325 | 1129.8  (882.7 - 1376.9) | 569370 | 3009 | 289.3  (239.7 - 338.8) | 282915 | 4871 | 1406.4  (1269.9 - 1542.8) |
|  | <65 years | 24456 | 223 | 734.6  (610.5 - 858.6) | 40876 | 229 | 366.1  (301.3 - 430.9) | 8787 | 85 | 717.4  (403.5 - 1031.2) | 123387 | 226 | 236.3  (173.1 - 299.4) | 67283 | 782 | 1330.1  (1156.1 - 1504) |
|  | ≥65 years | 82209 | 1579 | 1694.1  (1599 - 1789.3) | 130317 | 2810 | 1623.0  (1554.4 - 1691.6) | 63636 | 2240 | 2602.9  (2463.5 - 2742.4) | 445983 | 2783 | 478.5  (458.8 - 498.1) | 215632 | 4089 | 1678.9  (1623 - 1734.7) |
| 2018 | total | 118441 | 1947 | 894.5  (796.3 - 992.7) | 189434 | 3517 | 662.2  (605.5 - 718.9) | 82682 | 3195 | 1367.3  (1066.9 - 1667.6) | 592209 | 3038 | 256.8  (207.1 - 306.6) | 303277 | 5340 | 1386  (1242.7 - 1529.3) |
|  | <65 years | 25505 | 209 | 710.2  (586.8 - 833.6) | 41858 | 238 | 400.8  (330.6 - 471.1) | 9065 | 92 | 943.2  (560.8 - 1325.7) | 121864 | 195 | 204.9  (141.4 - 268.4) | 68238 | 773 | 1304.3  (1121.5 - 1487.2) |
|  | ≥65 years | 92936 | 1738 | 1553.0  (1467.1 - 1638.8) | 147576 | 3279 | 1595.6  (1530.4 - 1660.9) | 73617 | 3103 | 2881.7  (2742.1 - 3021.4) | 470345 | 2843 | 442.5  (423.7 - 461.2) | 235039 | 4567 | 1677.7  (1622.9 - 1732.6) |
| 2019 | total | 119234 | 1703 | 803  (706.8 - 899.1) | 191693 | 3030 | 453.5  (416.4 - 490.6) | 96126 | 3156 | 1412.9  (1031.6 - 1794.2) | 612475 | 2793 | 266.8  (214.9 - 318.8) | 326098 | 5213 | 1338.7  (1195.5 - 1482) |
|  | <65 years | 23494 | 182 | 651.1  (530.3 - 771.9) | 37631 | 158 | 221.8  (177.3 - 266.4) | 9225 | 100 | 1132.3  (645.5 - 1619.1) | 117520 | 191 | 237.9  (171.6 - 304.2) | 68336 | 730 | 1294.7  (1111.9 - 1477.4) |
|  | ≥65 years | 95740 | 1521 | 1345.4  (1261.6 - 1429.2) | 154062 | 2872 | 1280.9  (1222.4 - 1339.5) | 86901 | 3056 | 2414.9  (2289.7 - 2540.1) | 494955 | 2602 | 370.2  (353 - 387.4) | 257762 | 4483 | 1496.2  (1445.2 - 1547.2) |

Note: Standardized rates were calculated per 100,000 population for each specific disease. The rates were sex-age standardized using the 2019 Korean population aged 30 years and older as the standard population.

PPH: Potentially Preventive Hospitalization, COPD: Chronic Obstructive Pulmonary Disease, CHF: Congestive Heart Failure, HTN: Hypertension, DM: Diabetes Mellitus.
