# Supplementary material for: Age- and disability-based trends in potentially preventable hospitalizations: evidence from nationwide claims data in Korea
Source: Epidemiol Health. 2026 Feb 27;48:e2026012. doi: 10.4178/epih.e2026012 (PMC13219979; doi:10.4178/epih.e2026012)
Supplement: Supplementary Material 3. — Annual number of patients and sex-age standardized PPH rates among disabled individuals by age group and disease type (2010–2019) [file epih-48-e2026012-Supplementary-3.docx]

**Supplementary Material 3:** **Annual number of patients and sex-age standardized PPH rates among disabled individuals by age group and disease type (2010–2019)**

| Year | Age_  group | Asthma | | | COPD | | | CHF | | | HTN | | | DM | | |
| --- | --- | --- | --- | --- | --- | --- | --- | --- | --- | --- | --- | --- | --- | --- | --- | --- |
|  |  | No. of Patients | PPH ≥ 1 | Standardized rate (95% C.I.) | No. of Patients | PPH ≥ 1 | Standardized rate (95% C.I.) | No. of Patients | PPH ≥ 1 | Standardized rate (95% C.I.) | No. of Patients | PPH ≥ 1 | Standardized rate (95% C.I.) | No. of Patients | PPH ≥ 1 | Standardized rate (95% C.I.) |
| 2010 | total | 9691 | 209 | 2005.6  (1574.7 - 2436.5) | 15523 | 343 | 1755.5  (1463.8 - 2047.2) | 5011 | 272 | 3866.6  (2896.6 - 4836.5) | 48235 | 1015 | 2260.4  (2024.1 - 2496.6) | 26153 | 1760 | 8234.2  (7642.3 - 8826.1) |
|  | <65 years | 3523 | 67 | 1946.9  (1404.8 - 2488.9) | 5982 | 101 | 1560.5  (1197.3 - 1923.7) | 1339 | 52 | 3436.4  (2212.5 - 4660.3) | 18316 | 420 | 2333.6  (2034.6 - 2632.6) | 11099 | 884 | 8902.5  (8152.5 - 9652.5) |
|  | ≥65 years | 6168 | 142 | 2215.3  (1852.1 - 2578.4) | 9541 | 242 | 2452.0  (2143 - 2761) | 3672 | 220 | 5403.1  (4659.1 - 6147.1) | 29919 | 595 | 1999.0  (1837.5 - 2160.6) | 15054 | 876 | 5847.3  (5464.7 - 6230) |
| 2011 | total | 15421 | 349 | 1986.1  (1643.6 - 2328.5) | 24978 | 523 | 1283.6  (1105.9 - 1461.3) | 8315 | 389 | 4052.5  (3079.5 - 5025.6) | 76521 | 1057 | 1496.2  (1324.3 - 1668) | 41875 | 2270 | 6587.2  (6157.8 - 7016.6) |
|  | <65 years | 5449 | 104 | 1875.9  (1445.7 - 2306.1) | 9651 | 119 | 970.5  (752.9 - 1188.1) | 2184 | 87 | 3929.4  (2692.6 - 5166.2) | 28834 | 372 | 1519.0  (1301 - 1736.9) | 17343 | 1090 | 7090.4  (6546 - 7634.8) |
|  | ≥65 years | 9972 | 245 | 2379.5  (2078.8 - 2680.1) | 15327 | 404 | 2401.9  (2165.8 - 2638) | 6131 | 302 | 4492.2  (3967.6 - 5016.7) | 47687 | 685 | 1414.8  (1308 - 1521.6) | 24532 | 1180 | 4789.9  (4518.2 - 5061.5) |
| 2012 | total | 21431 | 495 | 2054.3  (1755.3 - 2353.3) | 35134 | 682 | 1230.8  (1083 - 1378.7) | 11171 | 505 | 4093.5  (3191.8 - 4995.2) | 101168 | 1151 | 1104.8  (974.6 - 1234.9) | 55130 | 2521 | 5175.3  (4845.8 - 5504.8) |
|  | <65 years | 7367 | 147 | 1957.5  (1581.6 - 2333.5) | 13354 | 156 | 947.2  (766 - 1128.3) | 2843 | 101 | 3976.3  (2829.5 - 5123.2) | 37358 | 358 | 1072.4  (907.6 - 1237.2) | 22358 | 1148 | 5451.7  (5034.5 - 5868.9) |
|  | ≥65 years | 14064 | 348 | 2399.7  (2143.3 - 2656.2) | 21780 | 526 | 2243.9  (2048.7 - 2439.1) | 8328 | 404 | 4512.0  (4048.4 - 4975.5) | 63810 | 793 | 1220.3  (1134.2 - 1306.4) | 32772 | 1373 | 4188.2  (3966.4 - 4410.1) |
| 2013 | total | 24731 | 500 | 2069.7  (1773.5 - 2366) | 40762 | 710 | 1179.5  (1035.7 - 1323.3) | 13787 | 543 | 3248.4  (2528.1 - 3968.8) | 122680 | 1219 | 951.1  (846.7 - 1055.6) | 67546 | 2925 | 4896  (4599.7 - 5192.3) |
|  | <65 years | 8498 | 171 | 2097.8  (1723.5 - 2472.1) | 15256 | 164 | 960.1  (782.2 - 1138) | 3522 | 98 | 3063.1  (2147.3 - 3978.8) | 44795 | 393 | 931.6  (799.4 - 1063.8) | 27013 | 1323 | 5161.1  (4785.8 - 5536.4) |
|  | ≥65 years | 16233 | 329 | 1969.7  (1751.6 - 2187.8) | 25506 | 546 | 1963.0  (1794.1 - 2132) | 10265 | 445 | 3910.6  (3527.7 - 4293.4) | 77885 | 826 | 1020.7  (950.2 - 1091.3) | 40533 | 1602 | 3949.3  (3755.2 - 4143.4) |
| 2014 | total | 31017 | 599 | 1758  (1515.7 - 2000.3) | 51509 | 884 | 1074.7  (958 - 1191.3) | 18065 | 684 | 3211.7  (2597.3 - 3826.2) | 151020 | 1315 | 769.2  (680.5 - 857.9) | 85005 | 3443 | 4392.7  (4145.3 - 4640.1) |
|  | <65 years | 10265 | 171 | 1690.2  (1384.9 - 1995.5) | 18651 | 203 | 855.8  (712 - 999.6) | 4486 | 119 | 3077.5  (2296.2 - 3858.8) | 53097 | 389 | 735.8  (623.5 - 848.1) | 32929 | 1488 | 4580.8  (4267.6 - 4894.1) |
|  | ≥65 years | 20752 | 428 | 2000.1  (1805.4 - 2194.7) | 32858 | 681 | 1856.3  (1713.1 - 1999.5) | 13579 | 565 | 3691.3  (3366.5 - 4016) | 97923 | 926 | 888.7  (830.4 - 946.9) | 52076 | 1955 | 3720.9  (3555.4 - 3886.4) |
| 2015 | total | 36688 | 720 | 1689.3  (1472.1 - 1906.6) | 60770 | 1125 | 1171.9  (1049.8 - 1293.9) | 22333 | 825 | 2590.8  (2114.4 - 3067.3) | 175455 | 1370 | 722.1  (635.2 - 808.9) | 101108 | 3667 | 4191.5  (3957.5 - 4425.4) |
|  | <65 years | 11544 | 189 | 1584.4  (1311 - 1857.7) | 21369 | 235 | 933.7  (782.3 - 1085) | 5362 | 135 | 2318.3  (1713.7 - 2922.8) | 60185 | 372 | 697.6  (587.4 - 807.8) | 38324 | 1600 | 4459.2  (4162.3 - 4756.1) |
|  | ≥65 years | 25144 | 531 | 2064.3  (1883.3 - 2245.3) | 39401 | 890 | 2022.6  (1884.4 - 2160.7) | 16971 | 690 | 3564.4  (3277.5 - 3851.3) | 115270 | 998 | 809.4  (758.1 - 860.8) | 62784 | 2067 | 3235.2  (3095 - 3375.4) |
| 2016 | total | 45380 | 860 | 1696.9  (1494.6 - 1899.1) | 76273 | 1352 | 1001.4  (905.9 - 1096.9) | 32735 | 1048 | 2138.7  (1772.2 - 2505.1) | 213645 | 1747 | 622.1  (557.2 - 686.9) | 124499 | 4204 | 3837.6  (3628 - 4047.2) |
|  | <65 years | 13664 | 240 | 1665.5  (1410 - 1921) | 25331 | 246 | 755.8  (638.1 - 873.5) | 7488 | 126 | 1842.2  (1377.6 - 2306.8) | 68394 | 414 | 562.4  (480.4 - 644.4) | 44459 | 1705 | 4044.5  (3778.5 - 4310.6) |
|  | ≥65 years | 31716 | 620 | 1808.9  (1660.7 - 1957.1) | 50942 | 1106 | 1878.7  (1761 - 1996.4) | 25247 | 922 | 3197.7  (2967.6 - 3427.9) | 145251 | 1333 | 835.2  (788.5 - 881.8) | 80040 | 2499 | 3098.6  (2974.9 - 3222.3) |
| 2017 | total | 52854 | 1032 | 1698.4  (1502.4 - 1894.3) | 90820 | 1547 | 876.2  (795.3 - 957.1) | 43406 | 1415 | 2044.6  (1717.8 - 2371.4) | 253305 | 1780 | 581.2  (510.4 - 652.1) | 149393 | 4671 | 3486.6  (3292.7 - 3680.5) |
|  | <65 years | 14187 | 272 | 1677.1  (1429.3 - 1925) | 27268 | 233 | 628.9  (529.5 - 728.3) | 9050 | 156 | 1773.0  (1358.4 - 2187.7) | 74491 | 386 | 550.0  (460 - 640.1) | 49393 | 1727 | 3644.7  (3398.4 - 3891) |
|  | ≥65 years | 38667 | 760 | 1774.3  (1637.1 - 1911.5) | 63552 | 1314 | 1759.6  (1655.7 - 1863.5) | 34356 | 1259 | 3014.5  (2820 - 3209) | 178814 | 1394 | 692.5  (653.3 - 731.7) | 100000 | 2944 | 2921.9  (2811.9 - 3031.9) |
| 2018 | total | 66113 | 1215 | 1475.6  (1299.3 - 1652) | 112752 | 1912 | 866.8  (785.4 - 948.3) | 54751 | 1813 | 1794.3  (1537.7 - 2050.8) | 301248 | 1872 | 460  (404.3 - 515.7) | 179713 | 5310 | 3354.2  (3174 - 3534.5) |
|  | <65 years | 15970 | 243 | 1396.9  (1173.9 - 1619.8) | 30947 | 262 | 637.6  (536.6 - 738.6) | 10483 | 165 | 1459.1  (1134.4 - 1783.8) | 81642 | 361 | 423.7  (353 - 494.3) | 55242 | 1888 | 3541.1  (3312 - 3770.2) |
|  | ≥65 years | 50143 | 972 | 1757.0  (1632.6 - 1881.4) | 81805 | 1650 | 1685.6  (1593 - 1778.2) | 44268 | 1648 | 2991.4  (2816.5 - 3166.3) | 219606 | 1511 | 589.8  (556.7 - 622.9) | 124471 | 3422 | 2687.0  (2590.9 - 2783) |
| 2019 | total | 72726 | 1241 | 1472.6  (1298.2 - 1646.9) | 124946 | 1963 | 751.4  (680.1 - 822.7) | 65101 | 2075 | 1831.4  (1569.4 - 2093.5) | 346721 | 1913 | 418.8  (366.2 - 471.5) | 209502 | 5647 | 3141  (2974.2 - 3307.9) |
|  | <65 years | 16791 | 267 | 1428.7  (1208 - 1649.3) | 31728 | 247 | 550.3  (462 - 638.6) | 11467 | 199 | 1573.1  (1240.7 - 1905.6) | 87803 | 345 | 391.7  (324.9 - 458.6) | 60475 | 1947 | 3332.9  (3120.7 - 3545.1) |
|  | ≥65 years | 55935 | 974 | 1629.3  (1510.6 - 1748.1) | 93218 | 1716 | 1469.6  (1388.1 - 1551) | 53634 | 1876 | 2754.1  (2596.3 - 2912) | 258918 | 1568 | 515.7  (486.3 - 545.1) | 149027 | 3700 | 2455.7  (2369.4 - 2542) |

Note: Standardized rates were calculated per 100,000 population for each specific disease. The rates were sex-age standardized using the 2019 Korean population aged 30 years and older as the standard population.

PPH: Potentially Preventive Hospitalization, COPD: Chronic Obstructive Pulmonary Disease, CHF: Congestive Heart Failure, HTN: Hypertension, DM: Diabetes Mellitus.
