# Supplementary material for: Age- and disability-based trends in potentially preventable hospitalizations: evidence from nationwide claims data in Korea
Source: Epidemiol Health. 2026 Feb 27;48:e2026012. doi: 10.4178/epih.e2026012 (PMC13219979; doi:10.4178/epih.e2026012)
Supplement: Supplementary Material 4. — Sex-age standardized rate of PPH including all disability types from 2010 to 2019 [file epih-48-e2026012-Supplementary-4.docx]

| **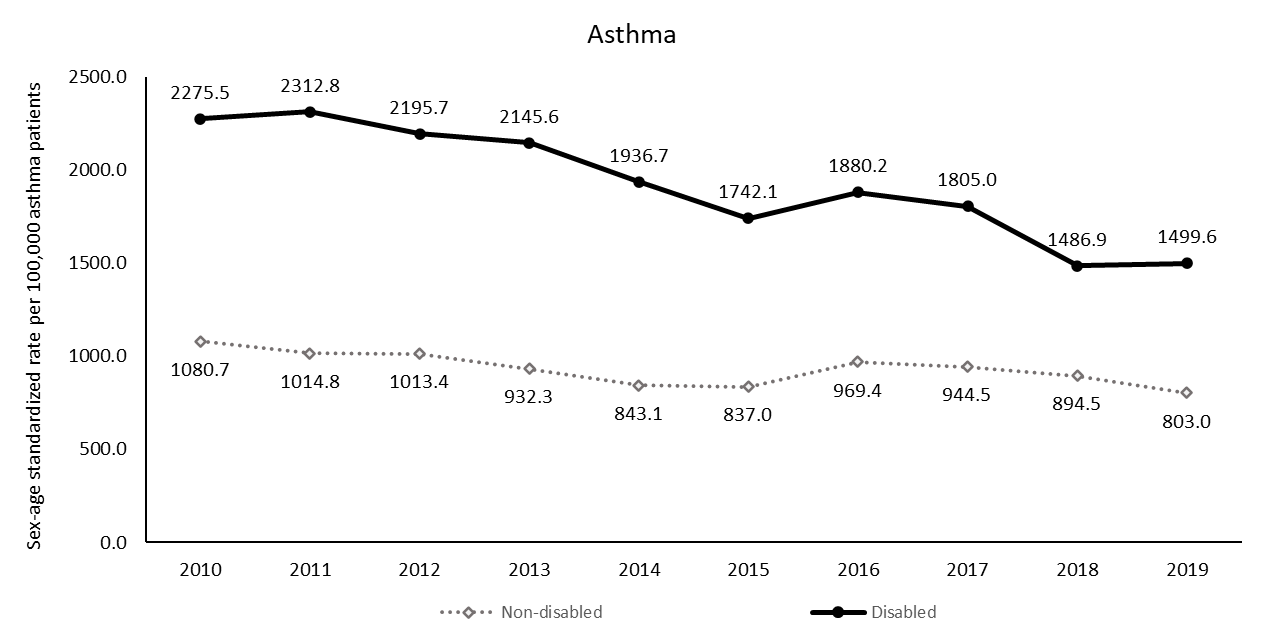** | **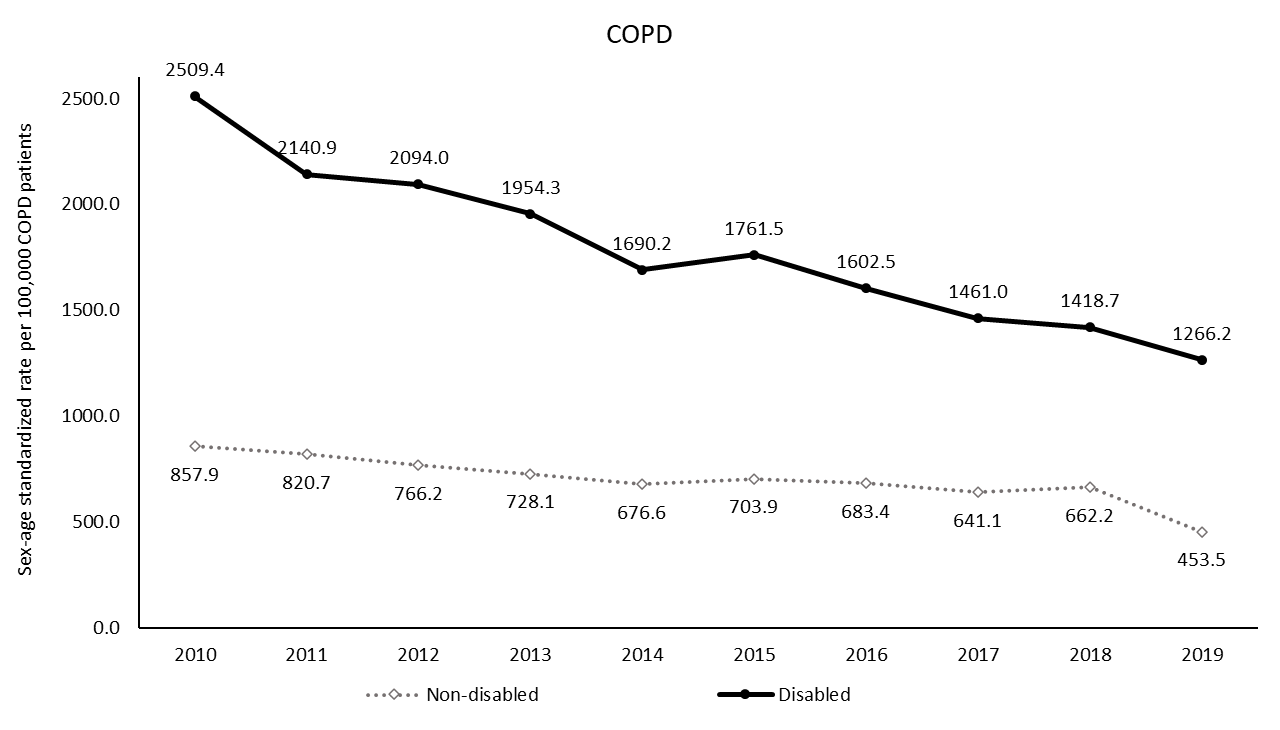** |
| --- | --- |
| **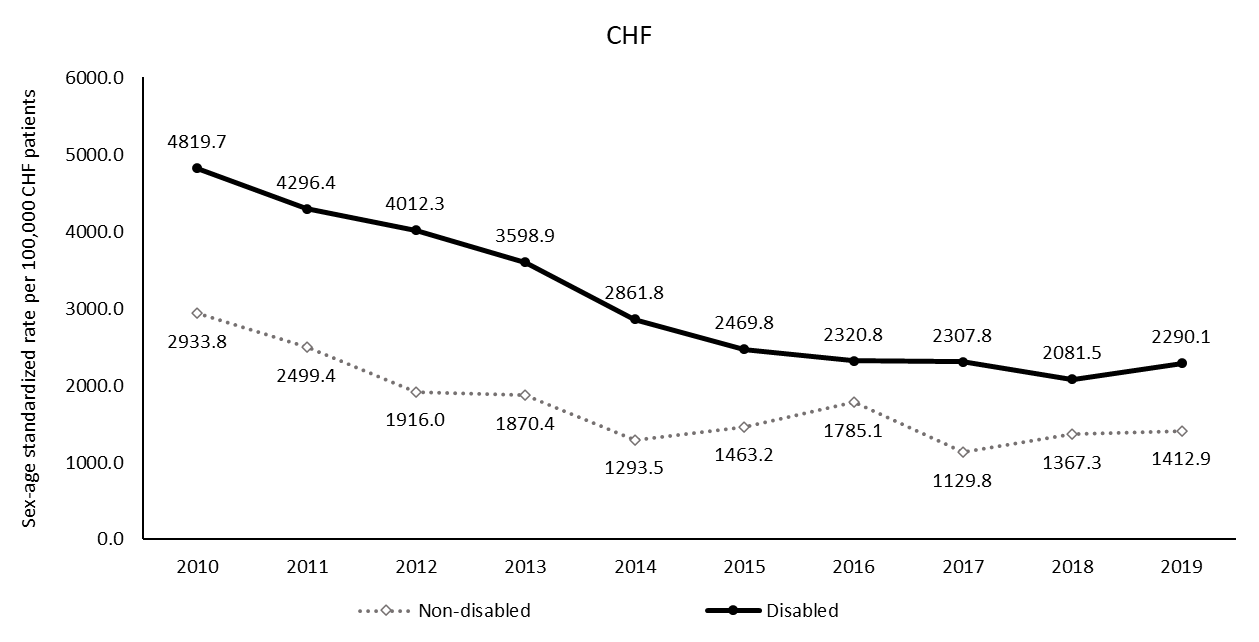** | **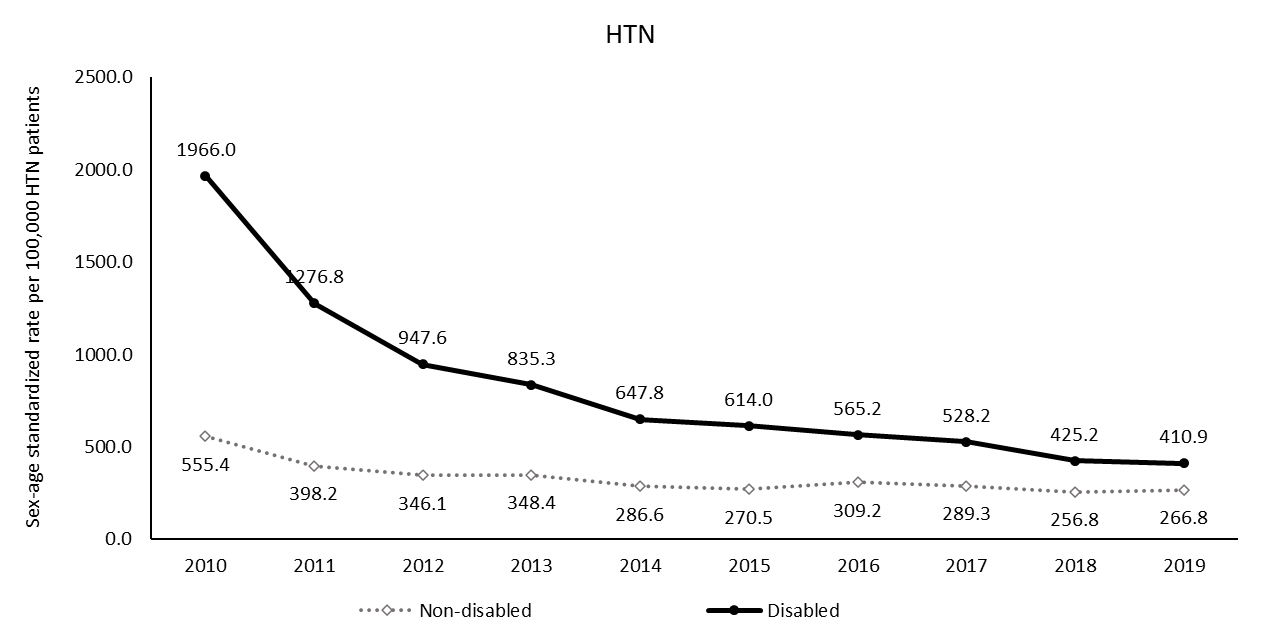** |
| **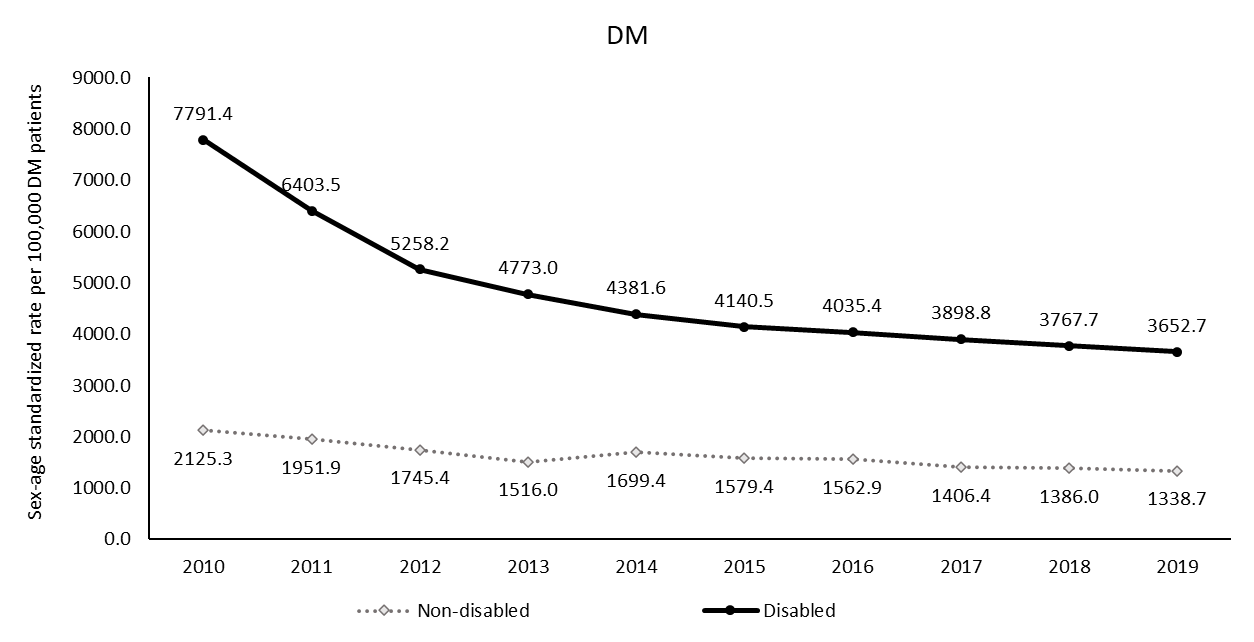** |  |

**Supplementary Material 4. Sex-age standardized rate of PPH including all disability types from 2010 to 2019**

Note: Standardized rates were calculated per 100,000 population for each specific disease. The rates were sex-age standardized using the 2019 Korean population aged 30 years and older as the standard population.

PPH: Potentially Preventive Hospitalization, COPD: Chronic Obstructive Pulmonary Disease, CHF: Congestive Heart Failure, HTN: Hypertension, DM: Diabetes Mellitus.
